# Single-Port Three-Dimensional Endoscopic-Assisted Axillary Lymph Node Dissection (S-P 3D E-ALND): Surgical Technique and Preliminary Results

**DOI:** 10.1155/tbj/6319218

**Published:** 2024-12-04

**Authors:** Chayanee Sae-Lim, Hung-Wen Lai, Papawee Chennavasin, Hsin-I Huang, Shih-Lung Lin, Ren-Hung Huang, Shou-Tung Chen, Dar-Ren Chen

**Affiliations:** ^1^Department of Surgery, Faculty of Medicine, Chulalongkorn University, Bangkok, Thailand; ^2^Endoscopic and Oncoplastic Breast Surgery Center, Changhua Christian Hospital, Changhua, Taiwan; ^3^Department of Surgery, Division of General Surgery, Changhua Christian Hospital, Changhua, Taiwan; ^4^Comprehensive Breast Cancer Center, Changhua Christian Hospital, Changhua, Taiwan; ^5^Minimal Invasive Surgery Research Center, Changhua Christian Hospital, Changhua, Taiwan; ^6^Kaohsiung Medical University, Kaohsiung, Taiwan; ^7^Division of Breast Surgery, Yuanlin Christian Hospital, Yuanlin, Taiwan; ^8^School of Medicine, Chung Shan Medical University, Taichung, Taiwan; ^9^School of Medicine, National Yang Ming Chiao Tung University, Taipei, Taiwan; ^10^Department of Surgery, Chulabhorb Hospital, Chulabhorn Royal Academy, Bangkok, Thailand; ^11^Department of Information Management, National Sun-Yat-Sen University, Kaohsiung, Taiwan; ^12^Department of Surgery, Division of Plastic and Reconstructive Surgery, Changhua Christian Hospital, Changhua, Taiwan; ^13^Department of Surgical Pathology, Changhua Christian Hospital, Changhua, Taiwan

**Keywords:** axillary lymph node dissection, breast cancer, endoscopic-assisted breast surgery (EABS), endoscopic axillary lymph node dissection, minimal-accessed breast surgery

## Abstract

**Background:** Endoscopic-assisted breast surgery (EABS) provides better cosmetic outcomes for breast cancer patients with small incisions in an inconspicuous area. However, an extended incision and heavy assistant retraction are usually required for an adequate exposure for conventional axillary lymph node dissection (ALND). Therefore, we propose an innovative single-port three-dimensional endoscopic-assisted ALND (S-P 3D E-ALND) to facilitate better visualization, and report its preliminary outcomes herein.

**Methods:** The surgical technique of the S-P 3D E-ALND, using either monopolar Endo Hook, LigaSure, or *Sonicision*, is described. A total of 11 breast cancer patients who received the S-P 3D E-ALND in a single institution from January 2023 to September 2023 were enrolled. The preliminary results of the S-P 3D E-ALND, including perioperative parameters, complication, and short-term oncological outcomes, were retrospectively analyzed.

**Results:** Endoscopic breast and axillary procedures were conducted via a single axillary incision. The primary success rate of the S-P 3D E-ALND was 100% without a conversion to open surgery. The median operative time for the S-P 3D E-ALND was 39 (IQR = 28, 49) minutes. Average blood loss during E-ALND was 3 (IQR = 3, 5) mL. The median number of harvested LN was 10 (IQR = 8, 11) LNs. During the median follow-up time of 7 months, there was no complication, lymphedema, shoulder stiffness, or chronic arm numbness found. None of the patients reported locoregional recurrence, distant metastasis, or mortality.

**Conclusion:** The S-P 3D E-ALND can serve as an alternative approach for ALND in breast cancer patients undergoing EABS, as our findings indicate it results in only minor complications.

**Summary**

• The S-P 3D E-ALND can serve as an alternative approach for ALND in breast cancer patients undergoing EABS, as our findings indicate it results in only minor complications.

## 1. Introduction

Endoscopic-assisted breast surgery (EABS) has been reported to provide better cosmetic outcomes [1–5] and comparable oncological results [1, 3, 6] for breast cancer patients compared with conventional surgery, using small incisions in inconspicuous areas such as the axilla or lateral chest. However, in patients with high-burden axillary lymph node (ALN) metastasis requiring axillary lymph node dissection (ALND), we often extend the EABS incision by 1-2 cm to access the deep and limited space of the axilla for the conventional ALND. This extension, along with the need for heavy assistant retraction and extensive surgical dissection to achieve adequate exposure [7–10], may compromise the benefits of small incisions in EABS and result in patient morbidities such as lymphedema or chronic arm numbness.

Compared with conventional ALND, multiple-incision two-dimensional (2D) endoscopic-assisted ALND has demonstrated acceptable numbers of harvested ALNs, lower postoperative pain, better shoulder range of motion, and noncompromised short- to medium-term oncological outcomes [7–14]. Therefore, we adapted the concept of our single-port air-insufflated breast cancer surgery [15] to develop the single-port three-dimensional endoscopic-assisted axillary dissection (S-P 3D E-ALND) performed via the single EABS incision without the need for wound extension. This approach aims to improve visualization of the deep axilla and increase the chances of preserving all neurovascular structures, thereby preventing the risk of arm morbidity. In addition, this technique can be an alternative for breast cancer patients undergoing EABS with high-burden ALN metastasis requiring ALND.

In this study, we aim to describe the surgical technique of the S-P 3D E-ALND using three different types of energy devices: monopolar Endo hook, or bipolar sealing instrument (LigaSure, Medtronic, Minneapolis, Minnesota, USA), or ultrasound energy device (*Sonicision*, Medtronic, Minneapolis, Minnesota, USA). We also report our preliminary results regarding perioperative outcomes, complications, and short-term oncological outcomes herein.

## 2. Materials and Methods

This study proposes the surgical technique of the S-P 3D E-ALND and reports its preliminary clinical outcomes. Breast cancer patients with pathological ALN metastasis undergoing the S-P 3D E-ALND between January 2023 to September 2023 at Changhua Christian Hospital (CCH), a tertiary medical center at central Taiwan, were included. The clinicopathological characteristics of patients including patient's age, body mass index, tumor histology and molecular subtypes, cancer staging, neoadjuvant treatment, and types of surgery were retrospectively retrieved. Perioperative parameters and postoperative outcomes were reviewed. All data were collected by specially trained nurses and confirmed by the principal investigator (HWL) subsequently.

The study was approved by the Institutional Review Board of the CCH (CCH IRB no. 230219). This report includes data and photos of several patients who had agreed and signed the consent for publication of their pictures at the outpatient clinic during the follow-up time when we started conducting this study.

### 2.1. Surgical Technique

#### 2.1.1. Indications for S-P 3D E-ALND

Patients are evaluated for eligibility for the S-P 3D EABS and E-ALND using preoperative breast sonography, mammography, and/or breast magnetic resonance imaging. In addition, liver sonography, chest x-ray, and whole-body bone scan are used to exclude the possibility of distant metastasis. Indications for the S-P 3D E-ALND are similar to the conventional ALND, including those with biopsy-proven ALN metastasis in clinically palpable ALNs or high ALN disease burden on ultrasonography or MRI, as well as clinically node-positive status after neoadjuvant systemic therapy. The S-P 3D E-ALND is offered to patients scheduled for the S-P 3D EABS as the therapeutic breast cancer surgery and is performed in the same operation following EABS.

#### 2.1.2. Sentinel Lymph Node Biopsy (SLNB) With Dual Tracers and Axillary Reverse Mapping

**Figure 1:**
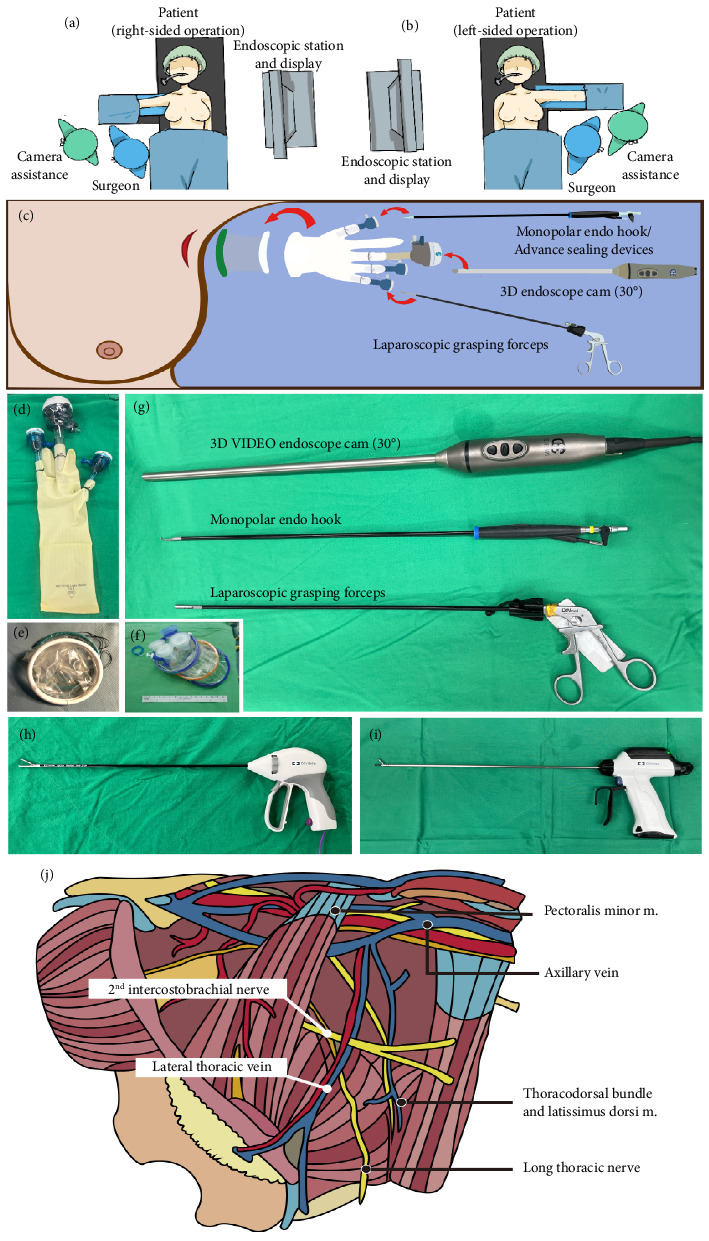
Illustrations and instruments of the single-port 3D endoscopic-assisted axillary lymph node dissection (S-P 3D E-ALND). (a) Operating layout for right-sided S-P 3D E-ALND: a surgeon and a camera assistance on the right side of patient's torso and endoscopic station and display on the left side of the patient. (b) Operating layout for left-sided S-P 3D E-ALND: a surgeon and a camera assistance on the left side of patient's torso and endoscopic station and display on the left side of the patient. (c) Insertion of a single port and endoscopic instruments: an energy device in 5 mm trocar on surgeon's right hand, laparoscopic grasping forceps in 5 mm trocar on surgeon's left hand, and 3D endoscope camera in a middle 12 mm trocar. (d) A glove-made single port made by a surgical glove, a 12 mm trocar, and two 5 mm trocars. (e) Alexis wound protector (*applied medical*, *Rancho Santa Margarita*, *California*, *USA*), a part of our glove-made single port. (f) A commercial Glove Port (Nelis, Gyeonggi-do, South Korea). (g–i) Endoscopic instruments used in 3D E-ALND. (j) Illustration of anatomical landmarks and neurovascular structures in left axillary cavity.

**Figure 2:**
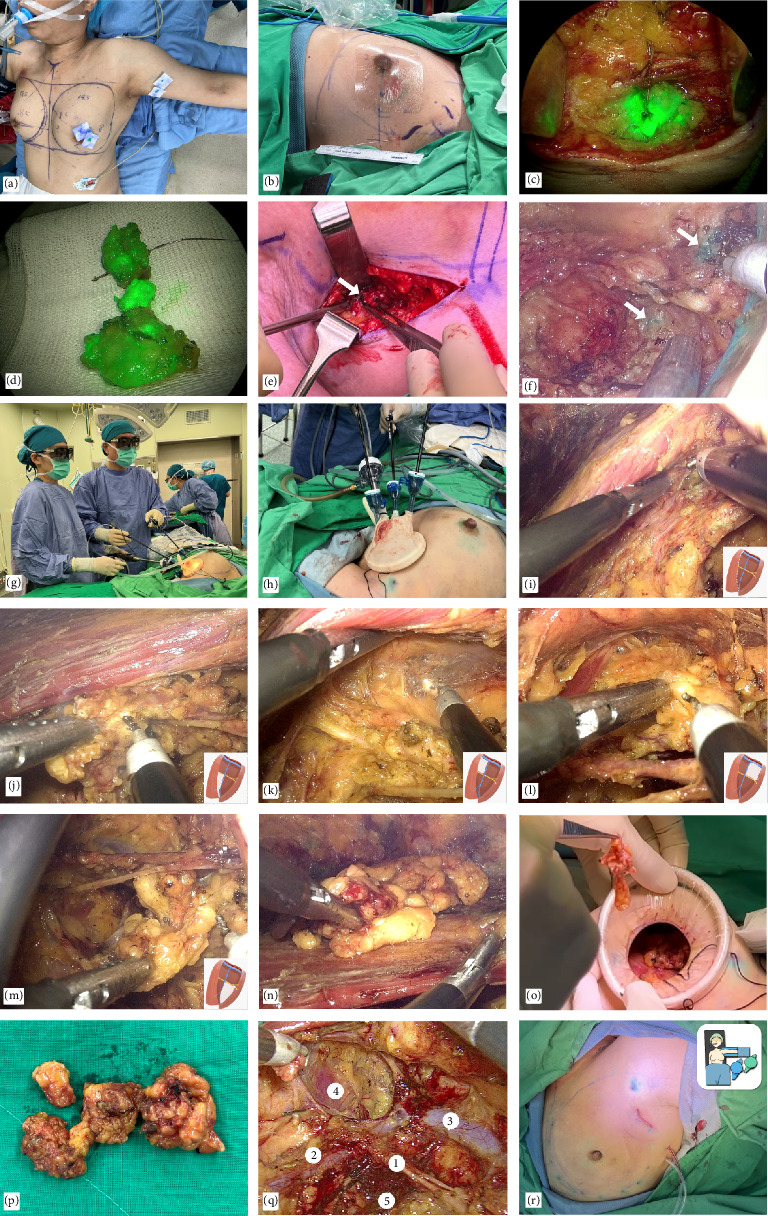
Surgical procedures of the single-port 3D endoscopic-assisted axillary lymph node dissection (S-P 3D E-ALND). (a) Patient's position for left S-P 3D E-ALND: supine, arm abduction to 90°, and shoulder elevation with surgical drapes to 30°. (b) 4 cm marked incision at left axilla. (c-d) ICG fluorescence of the sentinel nodes. (e-f) Blue-stained arm nodes seen under direct vision (e) and endoscopic view (f). (g) Intraoperative layout for left S-P 3D E-ALND. (h) Position of a single port during S-P 3D E-ALND. (i) Dissection along left pectoralis major border. (j) Removal of lower–inner group of axillary lymph nodes (ALNs). (k) Removal of upper–inner group of ALNs. (l) Removal of upper-outer group of ALNs. (m) Removal of lower–outer group of ALNs. (n) ALN specimens temporarily placed on pectoralis major muscle. (o) Removal of ALN specimens through an axillary incision. (p) ALN specimens. (q) Final appearance of axillary cavity after S-P 3D E-ALND; all preserved neurovascular structures (1 = the 2^nd^ intercostobrachial nerve, 2 = lateral thoracic vein, 3 = axillary vein, 4 = long thoracic nerve, and 5 = thoracodorsal neurovascular bundle). (r) An inconspicuous incision at left axilla after wound closure.

In the operating room, the patient is aligned at supine position with her arm abduction to 90° (Figures 1(a), 1(b), and 2(a)). We place surgical drapes underneath her scapula to elevate it to 30° (Figure 2(a)). We routinely conduct the SLNB with dual tracers, which include colloidal human serum albumin labeled with technetium-99m (Tc-99m) and indocyanine green (ICG), and combine with the axillary reverse mapping [16, 17] with patent blue dye. A 2.5–4 cm incision is created at lower axilla, over the location of the most intense radioactivity from Tc-99m indicating the location of sentinel nodes (Figures 1(c) and 2(b)). During the dissection, sentinel nodes displaying high radioactive signal and/or ICG fluorescence are removed (Figures 2(c) and 2(d)). If a blue-stained arm node is found, preservation of that node is considered to minimize the risk of postoperative lymphedema (Figures 2(e) and 2(f)). After removal of the SLNs, a gamma probe is used to confirm the absence of high residual radioactive signal in the axillary cavity. An intra-operative frozen section analysis of the SLNs is done. While awaiting the result, an EABS, either endoscopic-assisted breast-conserving surgery (E-BCS) or endoscopic-assisted nipple sparing mastectomy (E-NSM), is performed, which techniques are described in previous publications [4, 15, 18]. In case whose metastasis is identified in the SLNs meeting the criteria for ALND, the S-P 3D E-ALND is performed [19–21].

#### 2.1.3. S-P 3D E-ALND

The surgeon and camera assistance stand next to the patient's torso and the endoscopic station is set at the opposite side (Figures 1(a), 1(b), and 2(g)). After EABS, if the S-P 3D E-ALND is indicated, a glove-made single port (Figures 1(d), 1(e), and 2(h)) or a commercial port (Glove Port; Nelis, Gyeonggi-do, South Korea, Figure 1(f)) is reapplied. Our glove-made single port is created with *Alexis wound protector* (*Applied Medical, Rancho Santa Margarita, California, USA,*Figure 1(e)), a surgical glove, two 5-mm trocars, and a 12-mm trocar, which are arranged as in Figures 1(c) and 1(d). An optional 5-mm trocar may be added if additional grasper forceps are required to retract the pectoralis major border during the dissection (Figure 2(h)). After the port placement, carbon dioxide insufflation with the air pressure at 12 mmHg is applied to create a space for the S-P 3D E-ALND. A 30° 10-mm diameter camera TIPCAM 1 S 3D VIDEO Endoscope (KARL STORZ, Tuttlingen, Germany) is used for visualization (Figures 1(c) and 1(g)).

We perform the S-P 3D E-ALND using either one of the 3 different energy devices; monopolar Endo hook (Figure 1(g)), LigaSure (Figure 1(h)), or Sonicision (Figure 1(i)). The decision to use the certain energy device depends on the surgeon's experience and the patient's financial status. Specifically, in the initial phase, advanced energy sealing devices have been considered to control intraoperative bleeding. Later, once we became accustomed to the procedure or when patients could not afford the cost of advanced equipment, we performed the S-P 3D E-ALND with a monopolar Endo hook. All endoscopic instruments are demonstrated in Figure 1(d), 1(e), 1(f), 1(g), and 1(i).

When the S-P 3D E-ALND is performed, an axillary cavity is divided into four quadrants based on an intersection between lateral thoracic vein and the second intercostobrachial nerve (Figure 1(j)). We start the dissection at lateral border of pectoralis major muscle, which is the easiest part to access (Figure 1(i)). The dissection begins at a lower–inner quadrant (Figure 1(j)) and follows by upper–inner (Figure 1(k)), upper–outer (Figure 1(l)), and lower–outer quadrants (Figure 1(m)), respectively. An axillary content in each quadrant is subsequently removed to complete the standard level I and II ALND. The resected axillary specimens are temporarily placed above the pectoralis major muscle in E-NSM (Figure 1(n)) and in the glove part of the single port in E-BCS. Then, inflated gas is discontinued and a single port is taken off so that all axillary contents can be removed through an axillary incision (Figures 1(o) and 1(p)). The second intercostobrachial nerve, lateral thoracic vein, axillary vein, long thoracic nerve, and thoracodorsal neurovascular bundle are identified and preserved (Figure 1(q)). The wound is irrigated with warm distilled water and hemostasis is assured. Then, closed-suction drains are placed, and the incision is closed with Vicryl 3-0 and Monocryl 4-0 (Figure 1(r)).

The surgical technique of S-P 3D E-ALND is demonstrated in Figure 2 and Supporting Video 1 (monopolar Endo hook), Video 2 (*Sonicision*) and Video 3 (LigaSure).

### 2.2. Outcome Measures

The preliminary clinical outcomes of the S-P 3D E-ALND included operative time and blood loss, numbers of harvested ALN, length of hospital stay, perioperative complication, and short-term oncological safety. In addition, we compared operative time and blood loss from 3 different energy devices used in the S-P 3D E-ALND. Total operative time was defined as the operative time from skin incision to finishing the wound closure, including SLNB, the S-P 3D EABS, and E-ALND. The E-ALND time was defined as the operative time from the start of the S-P 3D E-ALND to the completion of the removal of all axillary specimens. Total intraoperative blood loss was counted during the whole operation, including SLNB, S-P 3D EABS, and E-ALND.

In addition, postoperative complications occurring within 3 months after surgery were recorded, including hematoma, seroma requiring aspiration, delayed wound healing, infection, and skin flap ischemia/necrosis. Arm morbidity was evaluated, in terms of lymphedema, arm numbness, and limited movement of the shoulder. Furthermore, oncologic outcomes were evaluated through the incidence of locoregional and distant recurrences and breast cancer-specific mortality. Incidence of recurrence and mortality due to breast cancer was ascertained at the most recent follow-up, which ended on 13 October 2023.

### 2.3. Statistical Analyses

Continuous variables were reported as the median and interquartile range. Categorical variables were expressed as numbers and percentages. The Kruskal–Wallis H-test was used for comparisons of intra-operative time and blood loss from three different devices. A two-tailed *p* value lower than 0.05 indicates statistical significance. All statistical analyses were performed with the statistical package SPSS (Version 22.0, SPSS, Chicago, IL, USA)

## 3. Results

### 3.1. Patient's Characteristics

A total of 11 breast cancer patients with pathological ALN metastasis undergoing the S-P 3D E-ALND between January 2023 to September 2023 at CCH was enrolled. The median age at the operation was 55.1 (IQR = 48.3, 59.5) years and the median BMI was 23.7 (IQR = 22.3, 26.9) kg/m^2^. In terms of clinical N staging, 7 patients (63.6%) have clinically positive ALN (cN+) and the remaining 4 patients (36.4%) were clinical node negative but later found macrometastases during SLNB. Eight patients (72.7%) were stage II–IV breast cancer and received neoadjuvant systemic therapy before the operation. Concurrent breast surgeries included 6 (54.5%) E-NSM and 4 (36.4%) E-BCS. One patient with occult breast cancer with axillary metastasis received only the S-P 3D E-ALND. The summary of patient characteristics is shown in Table 1.

### 3.2. Perioperative and Oncological Outcomes

Our success rate of the S-P 3D E-ALND was 100% without conversion to open surgery. There were 3 patients (27.2%) receiving the S-P 3D E-ALND using LigaSure (Figure 1(h)), 4 individuals (36.4%) using *Sonicision* (Figure 1(i)), and 4 patients (36.4%) using a monopolar Endo hook (Figure 1(g)). The median E-ALND time was 39 (IQR = 28, 49) minutes. An average overall operative duration was 265 (IQR = 210, 420) minutes, which were 217 (IQR = 181, 281) minutes for the S-P 3D E-BCS and E-ALND and 415 (IQR = 261, 523) minutes for the S-P 3D E-NSM, immediate reconstruction, and E-ALND. Total intraoperative blood loss was 30 (IQR = 20, 35) mL, and 3 (IQR = 3, 5) mL for E-ALND (Tables 2 and 3). Comparing the operative time and blood loss between three energy devices, we found no significant difference (Table 4).

The median number of harvested ALNs was 10 (IQR = 8, 11) nodes and the median number of ALN metastasis in the final pathology report was 1 (IQR = 1, 2) node. During the median follow-up time of 7 (IQR = 6, 9) months, none of the patients reported postoperative complication, in terms of hematoma, seroma, infection, skin flap ischemia/necrosis, and lymphedema. There were 2 patients (18.1%) reporting temporary decrease of sensation at medial arm and then improved within 3 months after the surgery. The full or almost full range of their shoulder mobility was preserved in all of the patients. No locoregional/distant recurrence or death was reported during the follow-up time. The clinical outcomes and short-term oncological safety of the 3D E-ALND are demonstrated in Table 2 and compared with previous studies in Table 3.

## 4. Discussion

In this study, we propose our new technique of the S-P 3D E-ALND and report preliminary results of this procedure. We enrolled 11 consecutive breast cancer patients with ALN metastasis undergoing the S-P 3D E-ALND. The clinical efficacy and short-term oncological outcomes of the S-P 3D E-ALND were retrospectively analyzed. The success rate of our S-P 3D E-ALND was 100%. None of the patients reported complication, total sensory loss of medial arm, or frozen shoulder during the median follow-up time of 7 months.

Despite the trend of de-escalating axillary surgery, NCCN guideline still recommends ALND for those with locally advanced or high ALN burden and residual nodal disease after neoadjuvant systemic therapy [21]. In our study, majority of the patients (72.7%) were in stage II–IV and had received neoadjuvant treatment (Table 1). Consequently, if micro- or macrometastasis was detected in a sentinel node during intraoperative frozen section analysis, ALND was indicated according to current standards of care [21]. This is reflected in our results, where the median number of metastatic ALNs identified was only 1 (Table 2).

Previously, when conventional ALND was required during the EABS, we faced challenges that necessitated extending the incision by 1-2 cm and relied heavily on assistant retraction to achieve adequate exposure in the deep and limited axillary space [7–10]. To address these challenges, we developed the S-P 3D E-ALND. This technique enhances visualization and minimizes the risk of neurovascular injury while allowing us to perform ALND effectively.

Another crucial indication for ALND is to establish adequate nodal status, which is essential for guiding adjuvant therapy, such as nodal irradiation [24] and the administration of CDK-4/6 inhibitors, given that nodal status remains a pivotal factor in treatment decisions [25, 26]. Therefore, the S-P 3D E-ALND can be an alternative in these settings.

From our results, the conversion rate to open surgery was 0, indicating the successful completion of the intended S-P 3D E-ALND procedure for all patients. The average operative time (E-ALND time) was comparable with those reported in previous studies [8–12, 14, 22] (Table 3). Intraoperative blood loss was counted for the entire operation as a routine practice in our center. However, we estimated minimal blood loss (3 [IQR = 3, 5] mL) during the specific period of S-P 3D E-ALND. These outcomes can be attributed to the high level of experience and expertise of the surgeons involved in our institution.

An advantage of gas inflation system and image magnification of 3D camera in the S-P 3D E-ALND allows for an adequate exposure of the operative field [15, 18, 27, 28] without the need for extending the incision nor assistant retraction, which is typically required in conventional ALND. Moreover, the image magnification system aids in the clear identification and preservation of all neurovascular structures, thereby reducing the risk of associated morbidities. As a result, none of the patients in our study experienced lymphedema, shoulder stiffness, or chronic arm numbness postoperatively.

In this study, we share our experience with 3 different energy devices; monopolar Endo hook, which rarely used in previous studies [8–12, 22] (Table 3), and advance sealing devices, LigaSure & *Sonicision*. Operative time and blood loss from three energy devices showed no significant difference (Table 4). From our experience, monopolar Endo hook appears to be effective to stop bleeding and can reduce the operative cost. In additionally, monopolar Endo hook is more available especially in low-middle income countries, which may facilitate an accessibility to this innovative technique. However, LigaSure and *Sonicision* seem to produce less blood loss and less drainage volume in breast and axillary surgery reported in previous studies [29, 30]. Therefore, we suggest using advance sealing devices in the patients who have a high risk of bleeding or high-output drainage.

Nevertheless, some limitations were considered in our study. First, our study was a preliminary report with a small sample size, which may restrict the generalizability of our findings. To address this limitation, we have been conducting a prospective study involving a larger cohort of patients to compare our results with conventional ALND and to evaluate patient satisfaction and aesthetic outcomes. Second, the median follow-up period of 7 months in our study may be considered relatively short for assessing medium- to long-term oncological safety. We have been extending the follow-up duration to gather more comprehensive and reliable long-term outcomes. Despite these limitations, we propose an innovative minimal-access technique for ALND using various energy devices and highlight its perioperative safety for patients undergoing EABS as therapeutic breast cancer surgery, aligning with the trend toward MABS.

## 5. Conclusions

The S-P 3D E-ALND can serve as an alternative approach for ALND in breast cancer patients undergoing EABS, as our findings indicate it results in only minor complications. However, we have been conducting further studies to gather long-term oncological outcomes and patient's satisfaction.

## Figures and Tables

**Table 1:** Characteristics of breast cancer patients undergoing the single-port three-dimensional endoscopic-assisted axillary lymph node dissection (S-P 3D E-ALND).

	All (*N* = 11)
Age, y, median (IQR)	55.1 (48.3, 59.5)
BMI, km/m^2^, median (IQR)	23.7 (22.3, 26.9)
Location, *N* (%)	
Left	5 (45.5)
Right	6 (54.5)
Clinical *N* stage, *N* (%)	
N0	4 (36.4)
N1	4 (36.4)
N2	3 (27.2)
Clinical staging, *N* (%)	
I	3 (27.3)
II	1 (9.1)
III	6 (54.5)
IV	1 (9.1)
Neoadjuvant treatments, *N* (%)	
Yes	8 (72.7)
No	3 (27.3)
Breast surgery, *N* (%)	
E-NSM	6 (54.5)
E-BCS	4 (36.4)
E-ALND only	1 (9.1)
Pathology, *N* (%)	
IDC	10 (90.9)
Solid papillary carcinoma	1 (9.1)
Pathology tumor size, cm, median (IQR)	2.3 (1.1, 3.2)
Pathology *N* staging, *N* (%)	
N0	0
N1	10 (90.9)
N2	0
N3	1 (9.1)
Pathological stage, *N* (%)	
I	0
II	9 (81.8)
III	1 (9.1)
IV	1 (9.1)
ER, *N* (%)	
Positive	9 (81.2)
Negative	2 (18.8)
PR, *N* (%)	
Positive	5 (45.5)
Negative	6 (54.5)
HER-2, *N* (%)	
Positive	3 (27.2)
Negative	8 (72.8)
Ki 67, *N* (%)	
≦ 14	6 (54.5)
> 14	5 (45.5)
Subtypes, *N* (%)	
Luminal A	6 (54.5)
Luminal B1	1 (9.1)
Luminal B2	2 (18.2)
HER-2	1 (9.1)
TNBC	1 (9.1)
Adjuvant treatments, *N* (%)	
Adjuvant hormonal therapy (NA = 1)	8 (72.7)
Adjuvant chemotherapy	10 (90.9)
Adjuvant radiation therapy	9 (81.8)

Abbreviations: DCIS = ductal carcinoma in situ, E-ALND = endoscopic axillary lymph node dissection, E-BCS = endoscopic breast conserving surgery, E-NSM = endoscopic nipple sparing mastectomy, ER = estrogen receptor, HER-2 = human epidermal growth factor receptor −2, IDC = invasive ductal carcinoma, NA = not available, PR = progesterone receptor, TNBC = triple negative breast cancer.

**Table 2:** Clinical outcomes of the single-port three-dimensional endoscopic-assisted axillary lymph node dissection (S-P 3D E-ALND).

	All (*N* = 11)
Success rate, *N* (%)	11 (100)
Dissection instruments, *N* (%)	
LigaSure	3 (27.2)
Sonicision	4 (36.4)
Monopolar endo hook	4 (36.4)
Operation time, minutes, median (IQR)	265 (210, 420)
E-NSM, reconstruction, and E-ALND	415 (261, 523)
E-BCS and E-ALND	217 (181, 281)
E-ALND time, minutes, median (IQR)	39 (28, 49)
E-ALND time in E-NSM cases	42 (22, 49)
E-ALND time in E-BCS cases	37 (30, 51)
Total intraoperative blood loss, median (IQR)	30 (20, 35)
Blood loss during E-ALND, ml, median (IQR)	3 (3, 5)
Hospital length of stay, days, median (IQR)	5 (4, 8)
Number of LN harvested, median (IQR)	10 (8, 11)
Numbers of ALN metastasis, median (IQR)	1 (1, 2)
Complications, *N* (%)	
Hematoma	0
Seroma requiring aspiration	0
Delayed wound healing	0
Infection	0
Skin flap ischemia/necrosis	0
Arm morbidity, *N* (%)	
Lymphedema	0
Temporary decrease of sensation at medial arm	2 (18.1)
Total sensory loss of medial arm	0
Limited shoulder ROM affecting daily activity	0
Event after the surgery, *N* (%)	
Breast recurrence	0
Axillary recurrence	0
Distant metastasis	0
Death	0
Follow up time, months, median (IQR)	7 (6, 9)

Abbreviations: E-BCS = endoscopic-assisted breast conserving surgery, E-NSM = endoscopic-assisted nipple sparing mastectomy, ROM = range of motion, SD = standard deviation.

**Table 3:** Published literature related to endoscopic axillary dissection.

Studies	Published journal	Patients (*N*)	Breast operations	Incisions, endoscope	Insufflation/retraction/suspension	Liposuction (Y/N)	E-ALND time (mins)	Blood loss	Harvested LNs (N)	Seroma (%)	Lymphedema (%)	Chronic arm numbness (%)	Shoulder stiffness (%)
						Coagulator (M/B/U)^a^							
Malur, Bechler, and Schneider [11]	Surgical Laparoscopy Endoscopy and Percutaneous Techniques	100	Open BCS	Multiple, anterior axilla, 2D 30° camera	CO2 inflation, 10 mmHg	N	75	20	16	4	1	14	2
						B							
Lim et al. [12]	The American Journal of Surgery	30	Open BCS	Multiple, periareolar and lateral chest, 2D 30° camera	Retraction	N	37	NR	15	43	0	3.3	Minimal
						M, B							
Fang et al. [10]	Surgical Oncology	32	Open BCS	Multiple, periareolar and lateral chest, 2D 30° camera	CO2 inflation, 6–8 mmHg	N	70.5	28.7	13.3	3.1	NR	25	Satisfied
						U							
Wu et al. [22]	Molecular and Clinical Oncology	106	Open TM	Multiple, periareolar and IMF, 2D 30° camera	CO2 inflation, 8 mmHg	N	85.5	28.7	12.2	0	0	0	0
						U							
Luo et al. [8, 9, 23]	Surgical Laparoscopy Endoscopy and Percutaneous Techniques, Mayo Clinic Proceedings	996	NR	Multiple, anterior, mid, posterior axillary line, 2D 30° camera	CO2 inflation, 8 mmHg	Y	40.6	12.8	17.6	4.8	0	5	2.4
						M							
Liu et al. [14]	Nature	126	Open BCS	Multiple, periareolar and lateral chest	Suspension or CO2 inflation, 10 mmHg	N	43–45	88–96	15–16	NR	0	3–4	NR
						M							
Sae-lim et al.	Current study	11	E-NSM, e-BCS	Single, lower axilla, 3D 30° camera	CO2 inflation, 12 mmHg	N	39	3	10	0	0	0	0
						M, B, U							

Abbreviations: BCS = breast conserving surgery, E-ALND = endoscopic axillary lymph node dissection, E-BCS = endoscopic-assisted breast conserving surgery, E-NSM = endoscopic-assisted nipple sparing mastectomy, IMF = inframammary fold, NR = not reported, TM = total mastectomy.

^a^M = monopolar coagulator, B = bipolar coagulator, U = ultrasound energy sealing devices.

**Table 4:** Perioperative outcomes of the single-port three-dimensional endoscopic-assisted axillary lymph node dissection (S-P 3D E-ALND) using 3 different energy devices.

All (*N* = 11)	Median (IQR)	*p* valuea
Total operative time (minutes)		0.564
LigaSure	200 (200, 474)	
Sonicision	263 (214, 390)	
Monopolar endo hook	257 (191, 373)	
E-ALND time (minutes)		0.155
LigaSure	47 (40, 47)	
Sonicision	43 (30, 54)	
Monopolar endo hook	31 (17, 31)	
Blood loss (mL)		0.435
LigaSure	5 (3, 5)	
Sonicision	3 (3, 4.5)	
Monopolar endo hook	4 (3, 5)	

^a^Kruskal–Wallis *H*-test.

## Data Availability

The datasets used and/or analyzed during the current study are available from the corresponding author upon reasonable request.

## Nomenclature

ALN: Axillary lymph node
ALND: Axillary lymph node dissection
E-BCS: Endoscopic-assisted breast-conserving surgery
EABS: Endoscopic-assisted breast surgery
E-NSM: Endoscopic-assisted nipple sparing mastectomy
MABS: Minimal-accessed endoscopic breast surgery
SLNB: Sentinel lymph node biopsy
S-P 3D E-ALND: Single-port three-dimensional endoscopic-assisted axillary lymph node dissection

## Supporting Information

Supporting Information 1 Supporting Video 1 Surgical technique of the single-port three-dimensional endoscopic-assisted axillary lymph node dissection (S-P 3D E-ALND) using monopolar Endo hook.

Supporting Information 2 Supporting Video 2 Surgical technique of the single-port three-dimensional endoscopic-assisted axillary lymph node dissection (S-P 3D E-ALND) using *Sonicision*).

Supporting Information 3 Supporting Video 3 Surgical technique of the single-port three-dimensional endoscopic-assisted axillary lymph node dissection (S-P 3D E-ALND) using LigaSure.
